# Gene-gene Interaction Analyses for Atrial Fibrillation

**DOI:** 10.1038/srep35371

**Published:** 2016-11-08

**Authors:** Honghuang Lin, Martina Mueller-Nurasyid, Albert V. Smith, Dan E. Arking, John Barnard, Traci M. Bartz, Kathryn L. Lunetta, Kurt Lohman, Marcus E. Kleber, Steven A. Lubitz, Bastiaan Geelhoed, Stella Trompet, Maartje N. Niemeijer, Tim Kacprowski, Daniel I. Chasman, Derek Klarin, Moritz F. Sinner, Melanie Waldenberger, Thomas Meitinger, Tamara B. Harris, Lenore J. Launer, Elsayed Z. Soliman, Lin Y. Chen, Jonathan D. Smith, David R. Van Wagoner, Jerome I. Rotter, Bruce M. Psaty, Zhijun Xie, Audrey E. Hendricks, Jingzhong Ding, Graciela E. Delgado, Niek Verweij, Pim van der Harst, Peter W. Macfarlane, Ian Ford, Albert Hofman, André Uitterlinden, Jan Heeringa, Oscar H. Franco, Jan A. Kors, Stefan Weiss, Henry Völzke, Lynda M. Rose, Pradeep Natarajan, Sekar Kathiresan, Stefan Kääb, Vilmundur Gudnason, Alvaro Alonso, Mina K. Chung, Susan R. Heckbert, Emelia J. Benjamin, Yongmei Liu, Winfried März, Michiel Rienstra, J. Wouter Jukema, Bruno H. Stricker, Marcus Dörr, Christine M. Albert, Patrick T. Ellinor

**Affiliations:** 1 National Heart Lung and Blood Institute’s and Boston University’s Framingham Heart Study, Framingham, MA, USA; 2 Section of Computational Biomedicine, Department of Medicine, Boston University School of Medicine, Boston, MA, USA; 3 Institute of Genetic Epidemiology, Helmholtz Zentrum München, German Research Center for Environmental Health, Neuherberg, Germany; 4 Department of Medicine I, Ludwig-Maximilians-University Munich, Munich, Germany; 5 DZHK (German Centre for Cardiovascular Research), partner site Munich Heart Alliance, Munich, Germany; 6 Icelandic Heart Association, Kopavogur, Iceland; 7 Faculty of Medicine, University of Iceland, Reykjavik, Iceland; 8 McKusick-Nathans Institute of Genetic Medicine, Johns Hopkins University School of Medicine, Baltimore, MD, USA; 9 Cleveland Clinic, Cleveland, OH, USA; 10 Department of Biostatistics, University of Washington, Seattle, WA, USA; 11 Department of Biostatistics, Boston University School of Public Health, Boston, MA, USA; 12 Department of Biostatistical Sciences, Public Health Sciences, Wake Forest School of Medicine, Winston-Salem, NC, USA; 13 Vth Department of Medicine, Medical Faculty Mannheim, Heidelberg University, Theodor-Kutzer-Ufer 1-3, 68167 Mannheim, Germany; 14 Cardiac Arrhythmia Service, Massachusetts General Hospital, Boston, MA, USA; 15 Harvard Medical School, Boston, MA, USA; 16 Department of Cardiology, University of Groningen, University Medical Center Groningen, Groningen, the Netherlands; 17 Department of Cardiology, Leiden University Medical Center, the Netherlands; 18 Department of Gerontology and Geriatrics, Leiden University Medical Center, Leiden, the Netherlands; 19 Department of Epidemiology, Erasmus MC - University Medical Center Rotterdam, Rotterdam, the Netherlands; 20 Department of Functional Genomics, Interfaculty Institute for Genetics and Functional Genomics, University Medicine and Ernst-Moritz-Arndt University Greifswald, Greifswald, Germany; 21 DZHK (German Centre for Cardiovascular Research), partner site Greifswald, Greifswald, Germany; 22 Division of Preventive Medicine, Brigham and Women’s Hospital, Boston MA, USA; 23 Cardiovascular Research Center, Massachusetts General Hospital, Boston, MA, USA; 24 Center for Human Genetic Research, Massachusetts General Hospital, Boston, MA, USA; 25 Department of Surgery, Massachusetts General Hospital, Boston, MA, USA; 26 Program in Medical and Population Genetics, Broad Institute, Cambridge, MA, USA; 27 Research Unit of Molecular Epidemiology, Helmholtz Zentrum München, German Research Center for Environmental Health, Neuherberg, Germany; 28 Institute of Human Genetics, Helmholtz Zentrum München - German Research Center for Environmental Health, Neuherberg, Germany; 29 Institute of Human Genetics, Technische Universität München, Munich, Germany; 30 National Institute on Aging, National Institutes of Health, Bethesda, MD, USA; 31 Epidemiological Cardiology Research Center, Wake Forest School of Medicine, Winston Salem, NC, USA; 32 Cardiovascular Division, Department of Medicine, University of Minnesota Medical School, Minneapolis, MN, USA; 33 Institute for Translational Genomics and Population Sciences (J.I.R.), Departments of Pediatrics and Medicine, LABioMed at Harbor-UCLA Medical Center, Torrance, CA, USA; 34 Cardiovascular Health Research Unit, Departments of Medicine, Epidemiology and Health Services, University of Washington, Seattle, WA, USA; 35 Group Health Research Institute, Group Health Cooperative, Seattle, WA, USA; 36 Mathematical and Statistical Sciences, University of Colorado, Denver, Denver, CO, USA; 37 Department of Gerontology and Geriatric Medicine, Wake Forest School of Medicine, Winston-Salem, NC, USA; 38 Institute of Health and Wellbeing, College of Veterinary, Medical and Life Sciences, University of Glasgow, United Kingdom; 39 Robertson Center for Biostatistics, University of Glasgow, United Kingdom; 40 Department of Epidemiology & Internal Medicine, Erasmus MC - University Medical Center Rotterdam, Rotterdam, the Netherlands; 41 Department of Medical Informatics, Erasmus MC - University Medical Center Rotterdam, the Netherlands; 42 Institute for Community Medicine, University Medicine Greifswald, Greifswald, Germany; 43 Department of Epidemiology, Rollins School of Public Health, Emory University, Atlanta, GA, USA; 44 Department of Epidemiology, Cardiovascular Health Research Unit, University of Washington, Seattle, WA, USA; 45 Section of Cardiovascular Medicine and Preventive Medicine, Department of Medicine, Boston University School of Medicine, Boston, MA, USA; 46 Department of Epidemiology, Boston University School of Public Health, Boston, MA, USA; 47 Department of Epidemiology & Prevention, Public Health Sciences, Wake Forest School of Medicine, Winston-Salem, NC, USA; 48 Synlab Academy, Synlab Services, GmbH P5,7, 68161 Mannheim, Germany; 49 Clinical Institute of Medical and Chemical Laboratory Diagnostics, Medical University of Graz, Graz, Austria; 50 Medical Clinic V (Nephrology, Hypertensiology, Rheumatology, Endocrinology, Diabetology), Medical Faculty Mannheim, University of Heidelberg, Mannheim, Germany; 51 Inspectorate of Health Care, Utrecht, the Netherlands; 52 Department of Internal Medicine B, University Medicine Greifswald, Greifswald, Germany

## Abstract

Atrial fibrillation (AF) is a heritable disease that affects more than thirty million individuals worldwide. Extensive efforts have been devoted to the study of genetic determinants of AF. The objective of our study is to examine the effect of gene-gene interaction on AF susceptibility. We performed a large-scale association analysis of gene-gene interactions with AF in 8,173 AF cases, and 65,237 AF-free referents collected from 15 studies for discovery. We examined putative interactions between genome-wide SNPs and 17 known AF-related SNPs. The top interactions were then tested for association in an independent cohort for replication, which included more than 2,363 AF cases and 114,746 AF-free referents. One interaction, between rs7164883 at the *HCN4* locus and rs4980345 at the *SLC28A1* locus, was found to be significantly associated with AF in the discovery cohorts (interaction OR = 1.44, 95% CI: 1.27–1.65, *P* = 4.3 × 10^–8^). Eight additional gene-gene interactions were also marginally significant (*P* < 5 × 10^–7^). However, none of the top interactions were replicated. In summary, we did not find significant interactions that were associated with AF susceptibility. Future increases in sample size and denser genotyping might facilitate the identification of gene-gene interactions associated with AF.

Atrial fibrillation (AF) is the most common cardiac arrhythmia, estimated to affect about 33.5 million individuals globally^1^. The heritability of AF, particularly lone AF, has long been established^2^,^3^,^4^,^5^,^6^,^7^,^8^. Over the past few years, genome-wide association studies (GWAS) have successfully identified more than a dozen genetic loci associated with AF susceptibility^9^,^10^,^11^,^12^,^13^,^14^. These loci include genes involved in cardiac signaling, cardiopulmonary development, and regulation of atrial action potential duration. However, all together, these loci still explain less than 5% of the heritability of AF^15^, whereas a large proportion of heritability remains unknown^16^,^17^.

Epistasis refers to the interaction of multiple genes that might pose joint genetic effects. Epistasis plays a ubiquitous role in disease predisposition, conferring an increased risk in addition to the main effects for many complex diseases, such as breast cancer^18^ and coronary heart disease^19^. Gene-gene interactions play important roles in regulating various biological events and cellular behaviors^20^,^21^. However, it remains unclear whether gene interactions contribute to the biological basis of AF.

The most straightforward approach to identifying interactions is to perform an exhaustive search of all the possible combinations of genetic variants and to test if any of them are significantly associated with AF. However, a major problem with such a comprehensive search is the huge computational burden. Assuming one million SNPs are genotyped in a typical GWAS, a complete search of a two-marker model would require testing 5 × 10^11^ pairs of SNPs. This number would further increase exponentially for multiple-SNP models. The cost of multiple testing corrections even in the 1 million marker scenario is extreme. For example, a Bonferroni correction requires *P* < 1 × 10^−13^ for significance in such a number of tests. As few SNP pairs will meet this threshold, false negatives are likely without massive sample sizes.

It has been suggested that at least one variant in significant gene-gene interactions tends to have a strong main effect^22^. We therefore sought to identify potential interactions between top AF susceptibility SNPs and other genome-wide variants in relation to AF by performing a meta-analysis of results from multiple studies.

## Results

In total, our study included 8,173 AF cases and 65,237 AF-free referents of European ancestry from 15 studies. The clinical characteristics of the study participants are shown in Table 1.

Supplemental Figure 1 presents Q-Q plots for the interaction p-values of genome-wide SNPs with each of the AF-associated variants. The effect of population stratification was negligible, with genomic control λ ranging from 0.98 to 1.01.

**Figure 1:**
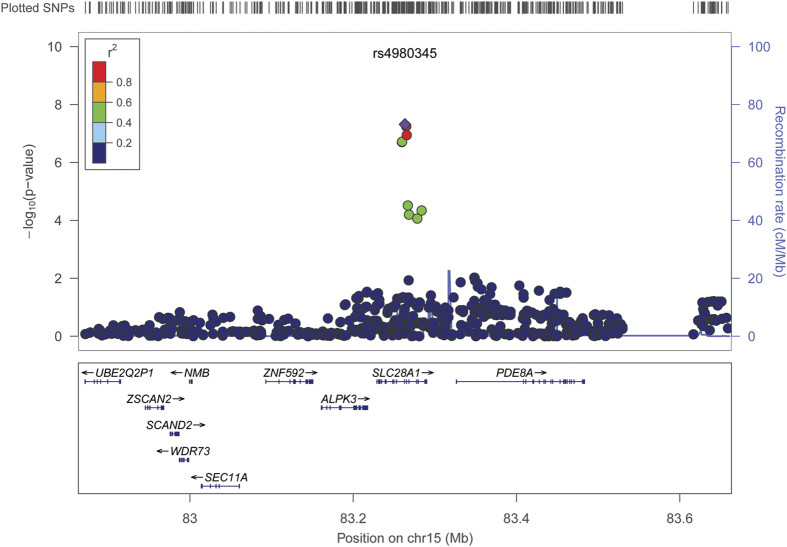
Regional plot of the interaction between rs7164883 and SNPs close to rs4980345. Each dot represents one SNP. The x-axis represents the chromosomal position, whereas the y-axis represents the −log_10_(*P*) of the association of the interaction between rs7164883 and each SNP with AF.

**Figure 2:**
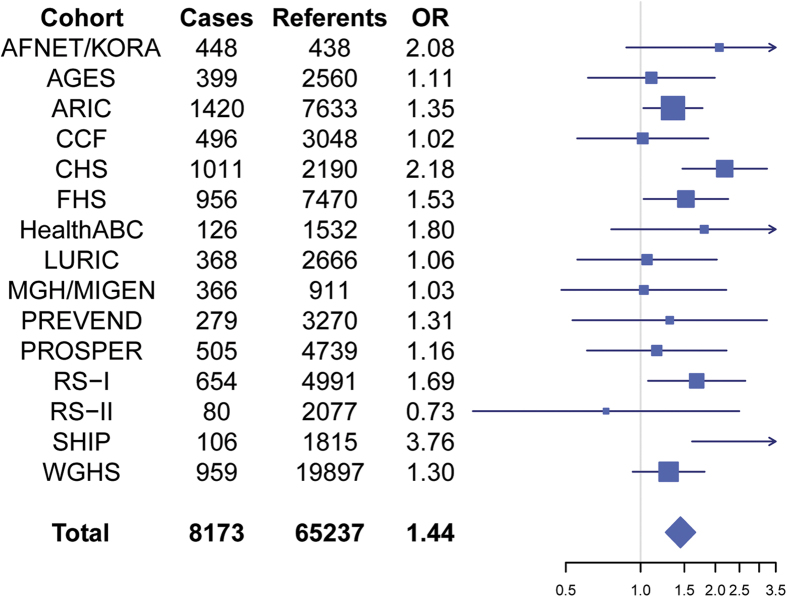
Forest plot of the association of interaction between rs7164883 and rs4980345 with AF in each study. Each line represents the 95% confidence interval, and the size is proportional to the number of cases. OR: odds ratio.

Table 2 shows the most significant interactions (*P* < 5 × 10^−7^) that were associated with AF susceptibility. The top 10 interactions for each AF SNP are shown in Supplemental Table 1. None of interactions reached the significance after adjusting for multiple testing (*P* < 5 × 10^−8^/17 = 2.8 × 10^−9^). Only one interaction, SNP rs7164883 with rs4980345 exceeded the traditional genome-wide significance threshold (*P* < 5 × 10^−8^) for association with AF (*P* = 4.3 × 10^−8^). Both interacting SNPs are located in chromosome 15, 12Mb apart. The corresponding regional plot is shown in Fig. 1, and the forest plot of each contributing study is shown in Fig. 2. The SNP rs7164883 is located within the first intron of *HCN4*, and was also one of the top SNPs found to be significantly associated with AF in our previous study^10^. The SNP rs4980345 was located within the tenth intron of *SLC28A1.* SNP rs4980345 was not associated with AF (*P* = 0.78) in marginal analyses from the prior meta-analysis^10^.

As shown in Table 2, eight additional interactions also showed suggestive association with AF (*P* < 5 × 10^−7^). These interactions included two each with rs12415501 (*NEURL*), rs3807989 (*CAV1*), and rs1448818 (*PITX2*). There was one marginally significant interaction each with rs10821415 (*C9orf3*) and rs2106261 (*ZFHX3*).

We also tested the association of rs2106261 at the *ZFHX3* locus and rs2200733 at the *PITX2* locus with AF, which was recently reported to be associated with AF in a meta-analysis of three Chinese samples (OR = 5.36, *P* = 8.0 × 10^−24^)^23^. The interaction, however, was not significant in any of the 16 studies included in the present paper, or in our meta-analysis (all with *P* > 0.05).

We then tried to replicate our findings in an independent cohort, UK Biobank, which included more than 2000 AF cases and 11,000 AF-free referents. As shown in Table 2, none of significant interactions from discovery phase were replicated (all with *P* > 0.05/9 = 0.0056).

## Discussion

In the past decade, increasing evidence has suggested that the genetic predisposition is an important factor that contributes to AF as well as many other cardiovascular diseases^24^,^25^. Due to the enormous number of association tests, few studies have been performed to investigate the associations of gene interactions with AF susceptibility. By restricting our analyses to interactions with known AF loci, we limited the multiple testing burden in our analysis and sought to examine the potential mechanisms by which variants at top loci contribute to AF susceptibility. One genome-wide significant gene interaction with AF, rs7164883 at the *HCN4* locus and rs4980345 at the *SLC28A1* locus, was found. Eight additional interactions were also marginally significant (*P* < 5 × 10^−7^), but did not withstand multiple testing correction. However, none of the top interactions were significant in the replication phase. It is noteworthy that the ORs of suggestive interactions from the replication cohort were very moderate. The most significant interaction from the discovery cohorts, rs7164883 with rs4980345, was even in the reverse direction in the replication cohort. Given that the replication cohort has similar genetic background to the discovery cohorts, the discrepancy indicates that these suggestive interactions are unlikely to be true AF-related interactions.

Our analyses were restricted to interactions with loci previously found to have a main effect association with AF. The underlying assumption of our approach is that interactions with significant effects tend to have observable main effects in at least one of the interacting SNPs^22^. However, it is possible that two variants without main effects might have large interaction effects. Our analysis will not identify such interactions. A variety of other methods have been developed to account for the enormous number of interactions between variants in genetic association studies^26^,^27^. One approach is to employ prior biological knowledge to limit the search space^28^. Gene interactions have been discovered through experimental assays. These might be used to guide the search of potential variant interactions. Additionally, it has been recognized that many known genetic interactions were enriched with well-studied pathways, and could only happen under certain conditions^29^, which might introduce additional bias to the analysis. In fact, none of the top interactions identified in the present study was reported in known interaction databases^30^, suggesting that the interaction between some variants may arise through some other intermediate pathway.

We did not detect a recently reported interaction with AF by Huang and colleagues^23^. This interaction involved rs2106261 at the *ZFHX3* locus and rs2200733 at the *PITX2* locus. SNP rs2106261 was the most significant SNP at the *ZFHX3* locus associated with AF in our earlier meta-analysis^9^. SNP rs2200733 was one of the top SNPs at the *PITX2* locus, and is in complete linkage disequilibrium (r^2^ = 1.0) with the most significant SNP rs6817105, the SNP we tested in this study. One possible explanation for the discrepancy between the findings of the two studies is the difference in allele frequency between the Asian population studied by Huang^23^ vs. the European ancestry population we studied (18% vs 28% for rs2106261, and 45% vs 16% for rs2200733, respectively). The effect of allelic difference and linkage disequilibrium could be amplified when the interaction was tested, suggesting that population stratification should be considered when comparing the results from studies based on different ethnicities.

We acknowledge several limitations of our study. All study participants in our study are of European ancestry, thus it is unclear whether our findings are relevant for populations of other ancestries. Furthermore, our analysis was restricted to two-variant interactions. However, it is possible that some interactions might involve more than two variants. Although our current study included more than 8,000 AF cases and 65,000 referents, it is possible that we did not have sufficient power to identify meaningful interactions for AF. We are currently expanding our AFGen Consortium to include additional cohorts, not only participants of European ancestry, but also participants of African ancestry and Asian ancestry. With the increasing sample size, we might be able to identify significant interactions in the future. In addition, we are currently imputing genotypes from individual studies to emerging reference panels such as the Haplotype Reference Consortium^31^, which is expected to provide better resolution to identify interacting variants. Given that our current study only tested interactions with known AF loci, we are also planning to expand our analyses to all interactions with the increasing sample size and more advanced computational methods.

In summary, we identified one genome-wide significant gene-gene interaction that was associated with AF susceptibility, suggesting that gene interactions might be involved in the development of AF. However, the finding was not replicated. Future work in functional genomics and efficient algorithms for epistasis analysis will likely facilitate the discovery of additional novel and high-order interactions that contribute to AF.

## Materials and Methods

### Study participants

Our discovery phase included individuals of European ancestry from 15 studies. These studies included the German Competence Network for Atrial Fibrillation/Cooperative Research in the Region of Augsburg (AFNET/KORA), Age, Gene/Environment Susceptibility Study (AGES) Reykjavik, Atherosclerosis Risk in Communities study (ARIC), Cleveland Clinic Lone AF GeneBank Study (CCAF), Cardiovascular Health Study (CHS), Framingham Heart Study (FHS), Health, Aging and Body Composition Study (HealthABC), Ludwigshafen Risk and Cardiovascular Health Study (LURIC), Massachusetts General Hospital Atrial Fibrillation Study (MGH), Prevention of Renal and Vascular Endstage Disease Study (PREVEND), the PROspective Study of Pravastatin in the Elderly at Risk study (PROSPER), Rotterdam Study (RS-I, RS-II), Study of Health in Pomerania (SHIP), and Women’s Genome Health Study (WGHS). The replication phase was performed on UK Biobank. The study protocol was approved by the internal review boards of Ludwig Maximilian University of Munich, University of Iceland, University of Minnesota, Cleveland Clinic, University of Washington, Boston University Medical Campus, Wake Forest School of Medicine, Heidelberg University, Massachusetts General Hospital, University Medical Center Groningen, Leiden University Medical Center, Erasmus MC - University Medical Center Rotterdam, University Medicine Greifswald, and Brigham and Women’s Hospital. The study was performed in accordance with the approved guidelines. All participants provided written informed consent to participate in genetic research.

### AF ascertainment

Details about AF ascertainment were described in previous publications^9^,^10^,^14^. Briefly, at each study, we combined evidence from a variety of sources to determine AF status, including electrocardiograms, Holter recordings, rhythm cards, medical records, and/or hospital discharge diagnostic codes. To achieve higher statistical power, we did not distinguish prevalent and incident AF cases, but combined them as individuals with a history of AF.

### Genotyping

Genotyping was performed independently in each study, using either Affymetrix SNP arrays or Illumina SNP arrays^9^, and then imputed to ~2.5 million SNPs in the HapMap II release 22 CEU panel to obtain a comprehensive set of SNPs across the genome. Detailed information regarding genotyping platforms, quality control metrics, and imputation methods for each study has been described previously^9^,^10^,^12^,^13^,^14^.

### Known AF-associated variants

The known AF-associated variants were selected from recent GWAS meta-analysis results^9^,^10^,^14^. Ellinor *et al*. reported nine AF loci in the meta-analysis of AF^9^. Sinner *et al*. reported five additional loci that were marginally significant in the earlier analysis but became genome-wide significant when combining with additional studies^10^. Lubitz *et al*. performed conditional analysis on the known *PITX2* locus^14^, and identified three additional independent SNPs within the locus. This resulted in a total of 17 AF-associated SNPs with genome-wide significance. The SNPs included one top SNP at each AF locus, and three additional independent SNPs at the *PITX2* locus. The full list of 17 SNPs is shown in Table 3.

### Statistical Analyses

A multivariable logistic regression model was used to test the associations of interacting SNPs with AF. Each interaction was comprised of one of the 17 AF SNPs, and one SNP from the ~2.5 million imputed HapMap Phase II SNPs. We assumed a multiplicative interaction effect as follows:


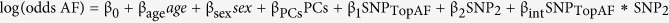


in which β_1_ and β_2_ are the main effects for the known AF SNP and the SNP to be tested, respectively. β_int_ represents the effect of the interaction between the AF SNP and the SNP to be tested. PCs represent principal components as necessary in each study to account for population structure. The model was also adjusted for *age* at DNA draw and *sex*, two factors that contribute significantly to AF risk. Studies with multiple study centers also adjusted for site. In order to account for the family correlation in FHS, we used generalized estimating equations (GEE) as implemented in the “geepack” R package. The association of each interaction with AF was adjusted for the independence working correlation structure in FHS, where each pedigree was a cluster in the robust variance estimate for the effect of interest.

The null hypothesis was that the interaction term, β_int_ = 0. Each study estimated and provided β_int_ and a robust estimate of standard error SE(β_int_) for each SNP interacting with each of the 17 AF-associated SNPs. Thus, we performed 17 interaction GWAS. The study-specific interaction regression parameter estimates r were then meta-analyzed using METAL^32^, applying a fixed effects approach weighted for the inverse of the variance. The effect of interaction was presented as an interaction odds ratio (OR), i.e., exp(*β*_*int*_). Given that we performed the genome-wide test for 17 SNPs, we defined significant interactions as those with a *P-*value less than 2.8 × 10^−9^ (=5 × 10^−8^/17 SNPs tested).

In the replication phase, we tested the association of significant or suggestive interactions (*P* < 5 × 10^−7^) in an independent cohort, UK Biobank. An interaction was replicated if it had the same direction of effect as the discovery, and the association *P* < 0.05/N, where N was the number of tests.

## Additional Information

**How to cite this article**: Lin, H. *et al*. Gene-gene Interaction Analyses for Atrial Fibrillation. *Sci. Rep.* **6**, 35371; doi: 10.1038/srep35371 (2016).

**Publisher’s note**: Springer Nature remains neutral with regard to jurisdictional claims in published maps and institutional affiliations.

## Supplementary Material

Supplementary Information

## Figures and Tables

**Table 1:** Clinical characteristics of the participating studies.

	Study	Group	n	Age, years	Men, %	HTN, %	BMI, kg/m^2^	Diabetes, %	MI, %	CHF, %
Discovery	AFNET/KORA	Cases	448	51.0 ± 7.6	68	41	28.1 ± 5.2	8	1	2
		Referents	438	55.8 ± 7.3	50	45	27.7± 4.3	4	0	1
	AGES	Cases	399	78.6±5.6	58	90	27.2 ± 4.4	13	6	9
		Referents	2,560	76.1±5.4	36	78	27.0 ± 4.5	11	5	1
	ARIC	Cases	1,420	56.8 ± 5.4	57	41	28.2 ± 5.3	13	9	8
		Referents	7,633	53.8 ± 5.6	45	24	26.8 ± 4.7	8	3	3
	CCF	Cases	496	58.8 ± 10.7	76	58	30.2 ± 6.2	6	0	8
		Referents	2,971	28.4 ± 22.2	38	–	–	–	–	–
	CHS	Cases	1,011	72.7 ± 5.4	44	62	26.4 ± 4.5	14	0	0
		Referents	2,190	72.0 ± 5.3	36	52	26.2 ± 4.4	11	0	0
	FHS	Cases	956	71.9 ± 12.3	57	68	28.3 ± 5.5	13	9	3
		Referents	7,470	51.8 ± 15.7	44	31	27.2 ± 5.3	5	2	1
	HealthABC	Cases	129	74.4 ± 2.9	63	87	26.3 ± 3.9	–	–	
		Referents	1,532	73.7 ± 2.8	52	62	26.6 ± 4.1	–	–	
	LURIC	Cases	361	66.4 ± 27.7	72	75	27.7 ± 4.2	45	33	30
		Referents	2,598	62.2 ± 10.7	70	73	27.4 ± 4.0	40	44	18
	MGH/MIGEN	Cases	366	53.4 ± 10.5	81	23	27.8 ± 5.0	3	1	3
		Referents	911	47.9 ± 8.8	53	–	–	–	–	–
	PREVEND	Cases	272	61.3 ± 9.4	67	68	27.7 ± 4.4	7	12	3
		Referents	3,277	48.4 ± 12.1	50	34	26.0 ± 4.2	4	2	0.1
	PROSPER	Cases	505	76.0 ± 3.5	58	64	27.1 ± 4.3	11	19	0
		Referents	4,739	75.3 ± 3.3	47	62	26.8 ± 4.2	10	13	0
	RS-I	Cases	954	72.6 ± 8.5	46	65	26.7 ± 3.7	17	11	14
		Referents	4,691	68.3 ± 8.8	40	53	26.2 ± 3.6	9	6	7
	RS-II	Cases	146	71.5 ± 9.7	54	78	27.2 ± 4.3	22	12	6
		Referents	2,011	64.3 ± 7.6	45	59	27.2 ± 4.2	12	4	1
	SHIP	Cases	106	62.0 ± 10.3	63	55	29.6 ± 5.1	23	13	27
		Referents	1,815	49.0 ± 14.4	47	24	27.2 ± 4.5	11	3	9
	WGHS	Cases	959	58.2 ± 7.6	—	40	27.2 ± 5.3	5	—	—
		Referents	19,897	53.9 ± 4.9	—	23	25.8 ± 4.9	2	—	—
Replication	UK Biobank	Cases	2,363	62.3 ± 5.8	70	52	29.1 ± 5.5	13	13	12
		Referents	114,746	56.7 ± 7.9	47	21	27.5 ± 4.8	5	2	0

HTN – hypertension, defined as systolic blood pressure ≥140 mmHg, or diastolic blood pressure ≥90 mmHg, or antihypertensive treatment. BMI – body mass index; Diabetes – diabetes mellitus; MI – myocardial infarction; CHF – heart failure. “−” Signifies data not available.

**Table 2:** Most significant interactions associated with AF (*P* < 5 × 10^−7^).

AF SNP		Interacting SNP							Interaction effects			Replication		
SNP	Closest gene	SNP	Locus	Closest gene	Location	Coding allele	CAF$	Meta *P* value	OR^+^	95% CI^*^	*P* value	OR^+^	95% CI^*^	*P* value
rs7164883	*HCN4*	rs4980345	15q25.3	*SLC28A1*	Intron	T	0.06	0.78	1.44	1.27–1.65	4.3 × 10^−8^	0.94	0.74–1.20	0.64
rs10821415	*C9orf3*	rs1492056	3p14.1	*MITF*	Intergenic	A	0.43	0.37	1.15	1.09–1.21	1.4 × 10^−7^	0.91	0.84–0.99	0.04
rs12415501	*NEURL*	rs699801	1p31.1	*CRYZ*	Intergenic	T	0.45	0.83	1.19	1.12–1.27	1.9 × 10^−7^	1.01	0.90–1.13	0.91
rs2106261	*ZFHX3*	rs12652090	5q34	*TENM2*	Intergenic	A	0.11	0.43	1.31	1.18–1.45	2.1 × 10^−7^	1.02	0.87–1.20	0.80
rs1448818	*PITX2 (2)*	rs693832	8p21.1	*MIR3622B*	Intergenic	C	0.11	0.36	1.27	1.16–1.39	3.1 × 10^−7^	1.03	0.89–1.19	0.72
rs3807989	*CAV1*	rs3802477	9q22.33	*GABBR2*	Intron	C	0.05	0.30	1.35	1.20–1.52	3.6 × 10^−7^	1.05	0.86–1.27	0.64
rs3807989	*CAV1*	rs2327995	6p22.3	*ATXN1*	Intron	G	0.27	0.74	1.15	1.09–1.22	4.3 × 10^−7^	1.05	0.95–1.15	0.32
rs1448818	*PITX2 (2)*	rs2328452	20p11.23	*RIN2*	Intergenic	G	0.88	0.92	1.25	1.15–1.37	4.7 × 10^−7^	0.94	0.82–1.08	0.37
rs12415501	*NEURL*	rs7946907	11p15.2	*SPON1*	Intron	A	0.54	0.05	1.18	1.11–1.26	4.7 × 10^−7^	1.07	0.96–1.20	0.23

^$^CAF: coding allele frequency; ^+^OR: odds ratio; ^*^CI: confidence interval.

**Table 3:** The list of 17 AF top SNPs. It includes four independent SNPs at the *PITX2* locus (with a number in parenthesis), and one top SNP at remaining 13 AF loci.

AF SNP	Closest Gene	Locus	Coding allele	Non-coding allele	Coding allele frequency	Source
rs6666258	*KCNN3*	1q21.3	C	G	0.3	13
rs3903239	*PRRX1*	1q24.2	G	A	0.45	9
rs4642101	*CAND2*	3p25.1	T	G	0.28	10
rs1448818	*PITX2 (2)*	4q25	C	A	0.17	14
rs6817105	*PITX2 (1)*	4q25	C	T	0.12	9,11
rs4400058	*PITX2 (3)*	4q25	A	G	0.12	14
rs6838973	*PITX2 (4)*	4q25	T	C	0.47	14
rs13216675	*GJA1*	6q22.31	C	T	0.27	10
rs3807989	*CAV1*	7q31.2	A	G	0.44	9
rs10821415	*C9orf3*	9q22.32	A	C	0.40	9
rs12415501	*NEURL*	10p14	T	C	0.11	10
rs10824026	*SYNPO2L*	10q22.2	G	A	0.14	9
rs6490029	*CUX2*	12q24.11	A	G	0.23	10
rs10507248	*TBX5*	12q24.21	G	T	0.23	10
rs1152591	*SYNE2*	14q23.2	A	G	0.45	9
rs7164883	*HCN4*	15q24.1	G	A	0.09	9
rs2106261	*ZFHX3*	16q22.3	T	C	0.16	9,12
