# Supplementary material for: Gene-gene Interaction Analyses for Atrial Fibrillation
Source: Sci Rep. 2016 Nov 8;6:35371. doi: 10.1038/srep35371 (PMC5099695; doi:10.1038/srep35371)
Supplement: Supplementary Information [file srep35371-s1.pdf]

Gene-gene Interaction Analyses for Atrial Fibrillation

Honghuang Lin, PhD[1,2]*; Martina Mueller-Nurasyid, PhD[3,4,5,$]; Albert V. Smith, PhD[9,10,$]; Dan E. Arking, PhD[12,$]; John Barnard, PhD[16,$]; Traci M. Bartz, MS[17,$]; Kathryn L. Lunetta, PhD[1,22,$]; Kurt Lohman, MSTAT[26,$]; Marcus E. Kleber, PhD[29,$]; Steven A. Lubitz, MD, MPH[31,32,$]; Bastiaan Geelhoed, PhD[37,$]; Stella Trompet, PhD[38,39,$]; Maartje N. Niemeijer, MD[42,$]; Tim Kacprowski, MSc[46,$]; Daniel I. Chasman, PhD[50,$]; Derek Klarin, MD[33,34,35,36,$]; Moritz F. Sinner, MD, MPH[4]; Melanie Waldenberger, PhD[5,6,29]; Thomas Meitinger, MD[5,7,8]; Tamara B. Harris, MD[11]; Lenore J. Launer, PhD[11]; Elsayed Z. Soliman, MD, MSc, MS[14]; Lin Y. Chen, MD, MS[15]; Jonathan D. Smith, PhD[16]; David R. Van Wagoner, PhD[16]; Jerome I. Rotter, MD[20]; Bruce M. Psaty, MD, PhD[18,19]; Zhijun Xie, MD, PhD[2]; Audrey E. Hendricks, PhD[1,23]; Jingzhong Ding, MD, PhD[27]; Graciela E. Delgado, MSc[29]; Niek Verweij, PhD[37]; Pim van der Harst, MD, PhD[37]; Peter W. Macfarlane, DSc[40]; Ian Ford, PhD[41]; Albert Hofman, MD, PhD[42]; André Uitterlinden, PhD[43]; Jan Heeringa, MD, PhD[42]; Oscar H. Franco, MD, PhD[42]; Jan A. Kors, PhD[45]; Stefan Weiss, PhD[46]; Henry Völzke, MD[47,49]; Lynda M. Rose, MS[50]; Pradeep Natarajan, MD, MMSc[32,33,34,36]; Sekar Kathiresan, MD[32,33,34,36†]; Stefan Kääb, MD, PhD[4,5†]; Vilmundur Gudnason, MD, PhD[9,10†]; Alvaro Alonso, MD, PhD[13†]; Mina K. Chung, MD[16†]; Susan R. Heckbert, MD, PhD[19,21†]; Emelia J. Benjamin, MD, ScM[1,24,25†]; Yongmei Liu, MD, PhD[28†]; Winfried März, MD[30,†]; Michiel Rienstra, MD, PhD[37,†]; J. Wouter Jukema, MD, PhD[38†]; Bruno H. Stricker, MMed, PhD[43,44†]; Marcus Dörr, MD[48,49†]; Christine M. Albert, MD, MPH[50†]; Patrick T. Ellinor, MD, PhD[31,32†]

Table of Contents

Supplemental	Title	Page
**S. Figure 1.**	Q-Q plots of interactions with AF	**3**
**S. Table 1.**	Top 10 interactions for each AF SNP	**6**
**Acknowledgement**		**11**

Supplemental Figure 1. QQ plots of interaction with AF

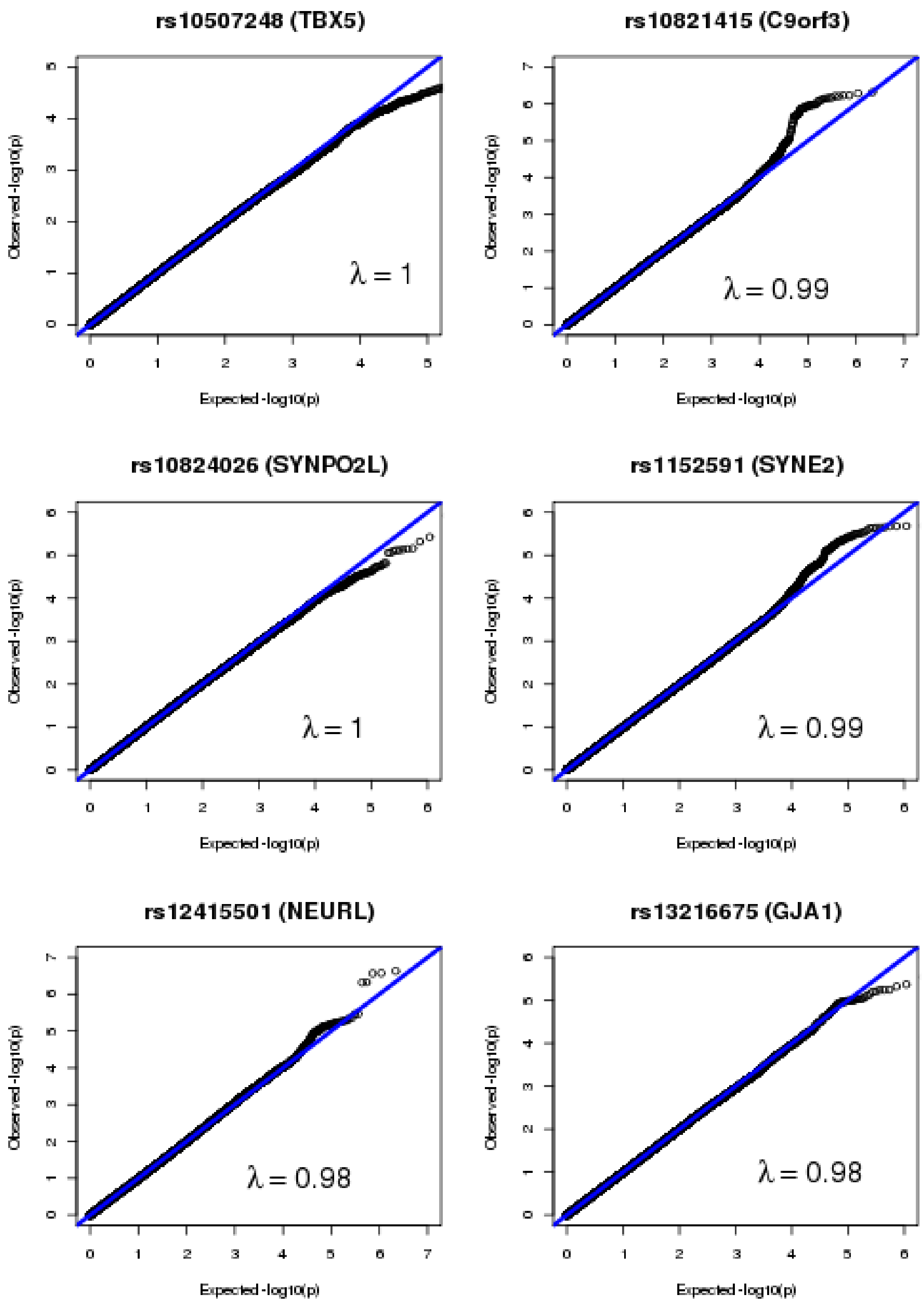

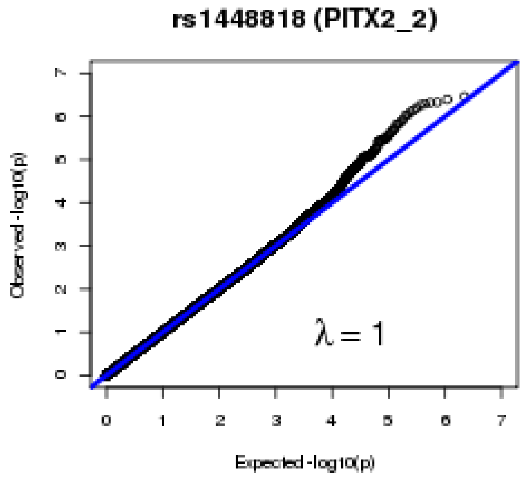
rs1448818 (PITX2_2)
λ = 1
Observed -log10(p)
Expected -log10(p)

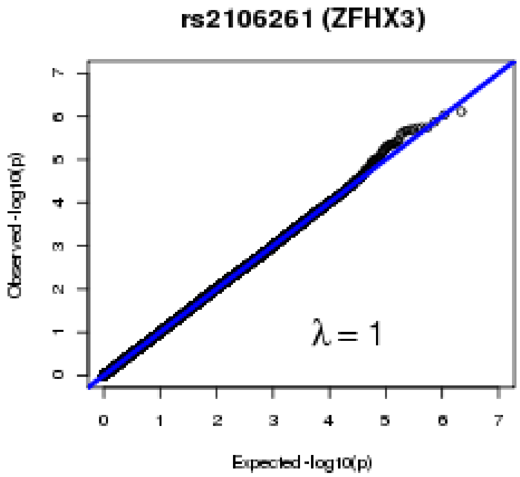
rs2106261 (ZFHX3)
λ = 1
Observed -log10(p)
Expected -log10(p)

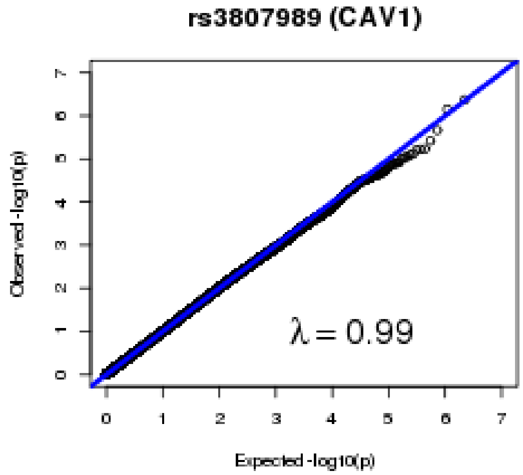
rs3807989 (CAV1)
λ = 0.99
Observed -log10(p)
Expected -log10(p)

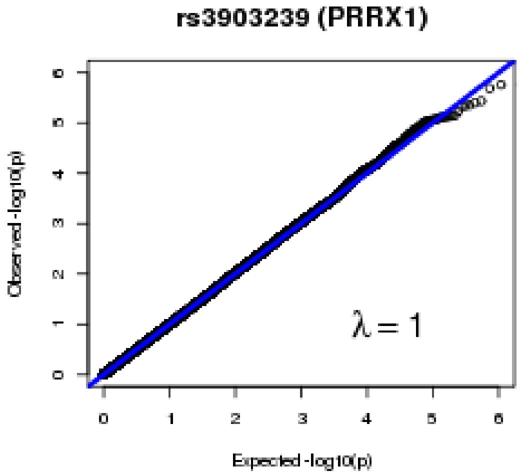
rs3903239 (PRRX1)
λ = 1
Observed -log10(p)
Expected -log10(p)

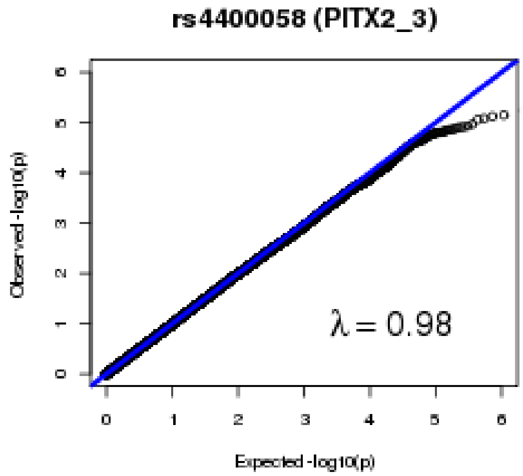
rs4400058 (PITX2_3)
λ = 0.98
Observed -log10(p)
Expected -log10(p)

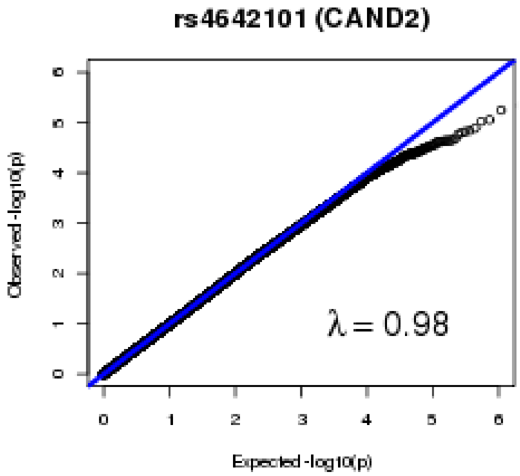
rs4642101 (CAND2)
λ = 0.98
Observed -log10(p)
Expected -log10(p)

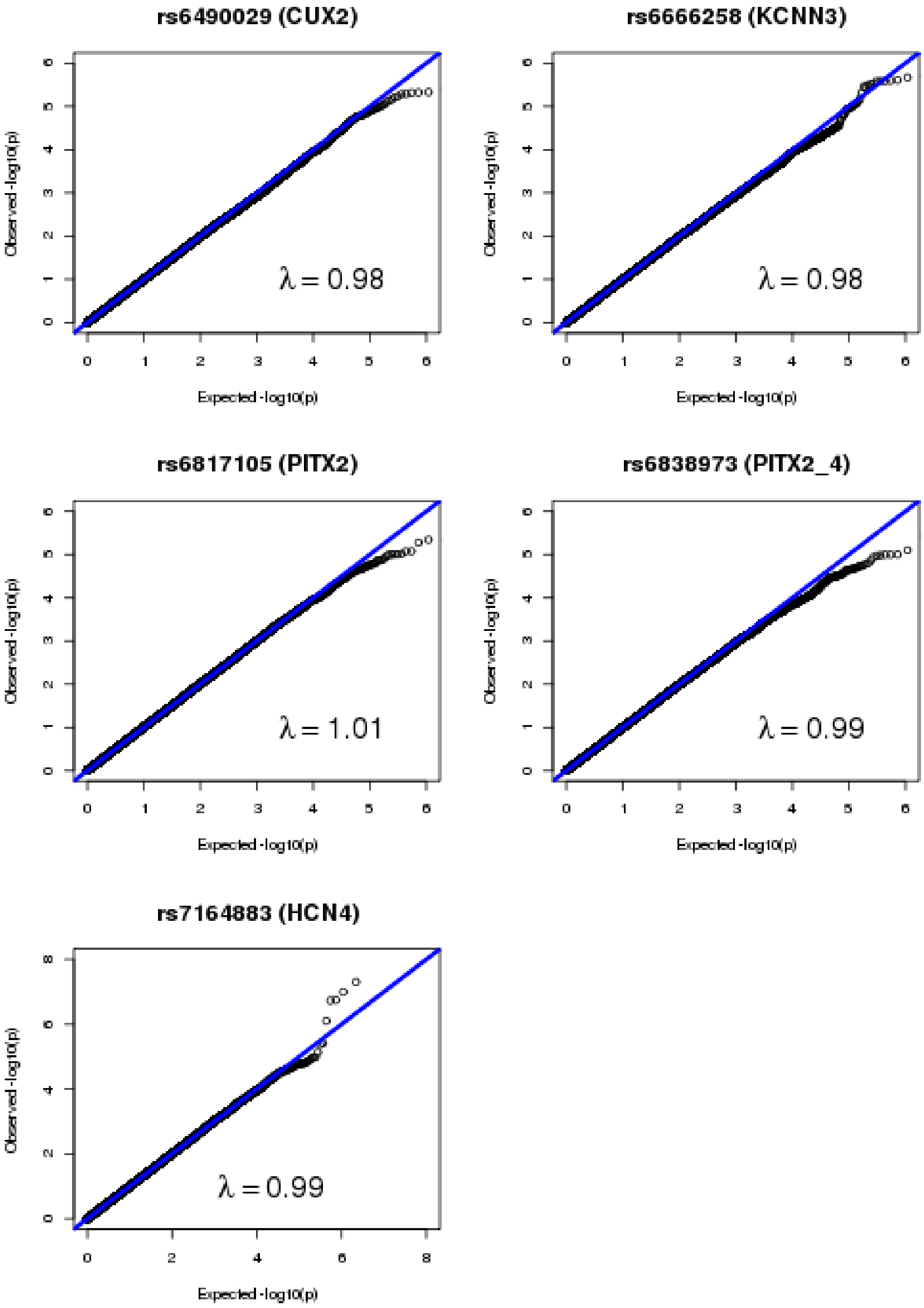
rs6490029 (CUX2)
λ = 0.98
rs6666258 (KCNN3)
λ = 0.98
rs6817105 (PITX2)
λ = 1.01
rs6838973 (PITX2_4)
λ = 0.99
rs7164883 (HCN4)
λ = 0.99
Observed -log10(p)
Expected -log10(p)

Supplemental Table 1. Top 10 interactions for each AF SNP

AF SNP		Interacting SNP						Interaction effects		
SNP	Closest gene	SNP	Locus	Closest gene	Location	Coding allele	CAF	OR+	95% CI*	*P* value
rs10507248	*TBX5*	rs10079502	5p14.1	*LOC340107*	Intergenic	T	0.94	1.50	1.26-1.80	8.2E-06
rs10507248	*TBX5*	rs2222738	2p16.3	*NRXN1*	Intergenic	A	0.70	0.87	0.82-0.93	1.3E-05
rs10507248	*TBX5*	rs1225886	20p12.1	*MACROD2*	Intron	T	0.41	1.14	1.07-1.20	1.3E-05
rs10507248	*TBX5*	rs1590042	2p16.3	*NRXN1*	Intergenic	A	0.16	1.19	1.10-1.28	1.4E-05
rs10507248	*TBX5*	rs10241686	7p21.1	*SNX13*	Intergenic	A	0.06	0.48	0.34-0.67	1.5E-05
rs10507248	*TBX5*	rs17683844	17q22	*TOM1L1*	Intergenic	T	0.81	1.19	1.10-1.28	1.5E-05
rs10507248	*TBX5*	rs1702510	18q23	*LOC339298*	Intergenic	T	0.80	1.17	1.09-1.25	2.0E-05
rs10507248	*TBX5*	rs1080669	19q13.33	*KLK13*	Intron	T	0.15	0.82	0.75-0.90	2.2E-05
rs10507248	*TBX5*	rs11770723	7p21.1	*SNX13*	Intergenic	T	0.94	2.04	1.47-2.84	2.3E-05
rs10507248	*TBX5*	rs4775500	15q22.2	*MGC15885*	Intergenic	T	0.23	1.15	1.08-1.22	2.3E-05
rs10821415	*C9orf3*	rs1492056	3p14.1	*MITF*	Intergenic	A	0.43	1.15	1.09-1.21	1.4E-07
rs10821415	*C9orf3*	rs6549264	3p14.1	*MITF*	Intergenic	A	0.55	0.88	0.83-0.92	4.8E-07
rs10821415	*C9orf3*	rs6480976	10q21.1	*MBL2*	Intergenic	A	0.57	0.87	0.83-0.92	5.1E-07
rs10821415	*C9orf3*	rs9839734	3p14.1	*MITF*	Intergenic	A	0.55	0.88	0.83-0.92	5.9E-07
rs10821415	*C9orf3*	rs9840397	3p14.1	*MITF*	Intergenic	A	0.55	0.88	0.83-0.92	5.9E-07
rs10821415	*C9orf3*	rs6772684	3p14.1	*MITF*	Intergenic	A	0.45	1.14	1.08-1.20	6.1E-07
rs10821415	*C9orf3*	rs9840590	3p14.1	*MITF*	Intergenic	A	0.55	0.88	0.83-0.92	6.2E-07
rs10821415	*C9orf3*	rs9834141	3p14.1	*MITF*	Intergenic	T	0.55	0.88	0.83-0.92	6.8E-07
rs10821415	*C9orf3*	rs7634340	3p14.1	*MITF*	Intergenic	A	0.46	1.14	1.08-1.20	6.8E-07
rs10821415	*C9orf3*	rs6549265	3p14.1	*MITF*	Intergenic	A	0.45	1.14	1.08-1.20	7.1E-07
rs10824026	*SYNPO2L*	rs7963529	12q12	*PRICKLE1*	Intergenic	A	0.89	1.36	1.21-1.54	8.3E-07
rs10824026	*SYNPO2L*	rs7302231	12q12	*PRICKLE1*	Intergenic	A	0.89	1.36	1.20-1.54	1.2E-06
rs10824026	*SYNPO2L*	rs8088466	18q12.3	*SLC14A2*	Intron	T	0.30	1.20	1.11-1.30	3.8E-06
rs10824026	*SYNPO2L*	rs6865647	5q11.2	*PDE4D*	Intron	A	0.87	0.79	0.71-0.87	4.8E-06
rs10824026	*SYNPO2L*	rs2054996	4p15.2	*PPARGC1A*	Intergenic	T	0.79	0.83	0.76-0.90	7.0E-06
rs10824026	*SYNPO2L*	rs6580059	5q33.2	*GALNT10*	Intron	T	0.85	1.28	1.15-1.43	7.2E-06
rs10824026	*SYNPO2L*	rs1950500	14q12	*NFATC4*	Intergenic	T	0.29	0.84	0.78-0.91	7.4E-06
rs10824026	*SYNPO2L*	rs12590407	14q12	*NFATC4*	Promoter	A	0.71	1.19	1.10-1.28	7.7E-06
rs10824026	*SYNPO2L*	rs2220642	4p15.2	*PPARGC1A*	Intergenic	A	0.79	0.83	0.76-0.90	7.7E-06
rs10824026	*SYNPO2L*	rs1511452	4p15.2	*PPARGC1A*	Intergenic	A	0.25	1.19	1.10-1.29	8.1E-06
rs1152591	*SYNE2*	rs11700857	21q22.3	*U2AF1*	Intergenic	T	0.84	0.84	0.79-0.90	1.8E-06
rs1152591	*SYNE2*	rs9812373	3q26.33	*USP13*	Intron	T	0.93	0.78	0.71-0.87	2.1E-06
rs1152591	*SYNE2*	rs17630775	7p12.3	*ABCA13*	Intron	A	0.06	0.72	0.63-0.83	2.1E-06
rs1152591	*SYNE2*	rs3852927	20q13.2	*CBLN4*	Intergenic	A	0.33	1.14	1.08-1.20	2.1E-06
rs1152591	*SYNE2*	rs9833938	3q26.33	*USP13*	Intron	T	0.93	0.79	0.71-0.87	2.1E-06
rs1152591	*SYNE2*	rs6069295	20q13.2	*CBLN4*	Intergenic	A	0.68	0.88	0.83-0.93	2.3E-06
rs1152591	*SYNE2*	rs13043784	20q13.2	*CBLN4*	Intergenic	T	0.66	0.88	0.84-0.93	2.3E-06
rs1152591	*SYNE2*	rs4785185	16q12.1	*ZNF423*	Intron	T	0.22	0.87	0.82-0.92	2.3E-06

AF SNP		Interacting SNP						Interaction effects		
SNP	Closest gene	SNP	Locus	Closest gene	Location	Coding allele	CAF	OR[+]	95% CI[*]	*P* value
rs1152591	*SYNE2*	rs8125772	20q13.2	*CBLN4*	Intergenic	A	0.68	0.88	0.83-0.93	2.4E-06
rs1152591	*SYNE2*	rs12481038	20q13.2	*CBLN4*	Intergenic	T	0.68	0.88	0.83-0.93	2.4E-06
rs12415501	*NEURL*	rs699801	1p31.1	*CRYZ*	Intergenic	T	0.45	1.19	1.12-1.27	1.9E-07
rs12415501	*NEURL*	rs3113980	1p31.1	*CRYZ*	Intergenic	T	0.45	1.19	1.11-1.27	2.4E-07
rs12415501	*NEURL*	rs1340985	1p31.1	*CRYZ*	Intergenic	A	0.55	0.84	0.79-0.90	2.7E-07
rs12415501	*NEURL*	rs3098953	1p31.1	*CRYZ*	Intergenic	T	0.55	0.84	0.79-0.90	2.7E-07
rs12415501	*NEURL*	rs7946907	11p15.2	*SPON1*	Intron	A	0.54	1.18	1.11-1.26	4.7E-07
rs12415501	*NEURL*	rs2303973	11p15.2	*SPON1*	Missense	A	0.54	1.18	1.11-1.26	4.8E-07
rs12415501	*NEURL*	rs11711838	3q28	*FGF12*	Intron	T	0.31	0.84	0.79-0.91	3.4E-06
rs12415501	*NEURL*	rs10164009	18p11.32	*METTL4*	Intergenic	T	0.93	0.69	0.59-0.81	3.5E-06
rs12415501	*NEURL*	rs10958142	8q21.13	*RALYL*	Intergenic	T	0.29	1.19	1.11-1.28	4.3E-06
rs12415501	*NEURL*	rs7833584	8q21.13	*RALYL*	Intergenic	T	0.29	1.19	1.11-1.28	4.4E-06
rs13216675	*GJA1*	rs1559765	11q24.3	*ETS1*	Intergenic	C	0.69	1.16	1.09-1.23	1.8E-06
rs13216675	*GJA1*	rs11221175	11q24.3	*ETS1*	Intergenic	C	0.69	1.16	1.09-1.23	2.8E-06
rs13216675	*GJA1*	rs4877276	9q21.33	*NTRK2*	Intergenic	T	0.45	1.14	1.08-1.21	4.3E-06
rs13216675	*GJA1*	rs17778070	4q31.23	*NR3C2*	Intergenic	T	0.80	0.85	0.80-0.91	4.8E-06
rs13216675	*GJA1*	rs11221187	11q24.3	*ETS1*	Intergenic	A	0.70	1.15	1.08-1.22	5.7E-06
rs13216675	*GJA1*	rs12567590	1q42.13	*PRSS38*	Intergenic	A	0.91	0.76	0.68-0.86	5.7E-06
rs13216675	*GJA1*	rs12562786	1q42.13	*PRSS38*	Intergenic	C	0.91	0.77	0.68-0.86	5.8E-06
rs13216675	*GJA1*	rs10269328	7p15.3	*RAPGEF5*	Intergenic	T	0.81	0.85	0.79-0.91	6.2E-06
rs13216675	*GJA1*	rs1940365	11q24.3	*ETS1*	Intergenic	A	0.53	1.14	1.07-1.20	6.3E-06
rs13216675	*GJA1*	rs11221163	11q24.3	*ETS1*	Intergenic	A	0.53	1.13	1.07-1.20	7.3E-06
rs1448818	*PITX2_2*	rs693832	8p21.1	*MIR3622B*	Intergenic	T	0.89	0.79	0.72-0.86	3.1E-07
rs1448818	*PITX2_2*	rs17469423	8p21.1	*MIR3622A*	Intergenic	A	0.11	1.27	1.16-1.39	3.5E-07
rs1448818	*PITX2_2*	rs10216643	8p21.1	*CCDC25*	Intergenic	A	0.90	0.79	0.72-0.87	4.0E-07
rs1448818	*PITX2_2*	rs2328452	20p11.23	*RIN2*	Intergenic	A	0.12	0.80	0.73-0.87	4.7E-07
rs1448818	*PITX2_2*	rs9653629	20p11.23	*RIN2*	Intergenic	A	0.12	0.80	0.74-0.87	4.8E-07
rs1448818	*PITX2_2*	rs11984514	8p21.1	*CCDC25*	Intergenic	T	0.10	1.26	1.15-1.38	5.2E-07
rs1448818	*PITX2_2*	rs17385257	8p21.1	*CCDC25*	Intergenic	T	0.10	1.26	1.15-1.38	5.5E-07
rs1448818	*PITX2_2*	rs17370440	20p11.23	*RIN2*	Intergenic	A	0.09	0.78	0.71-0.86	6.4E-07
rs1448818	*PITX2_2*	rs17298185	20p11.23	*RIN2*	Intergenic	A	0.09	0.78	0.71-0.86	7.3E-07
rs1448818	*PITX2_2*	rs805559	20q11.23	*RALGAPB*	Intron	A	0.17	1.21	1.12-1.30	7.9E-07
rs2106261	*ZFHX3*	rs12652090	5q34	*TENM2*	Intergenic	A	0.11	1.31	1.18-1.45	2.1E-07
rs2106261	*ZFHX3*	rs11640649	16q23.2	*CMIP*	Intron	T	0.06	1.54	1.30-1.83	7.7E-07
rs2106261	*ZFHX3*	rs16952029	16q23.2	*MAF*	Intergenic	A	0.18	1.26	1.15-1.38	9.1E-07
rs2106261	*ZFHX3*	rs4677777	3q29	*XXYLT1*	Intergenic	A	0.27	1.19	1.11-1.28	1.4E-06
rs2106261	*ZFHX3*	rs2160523	12p13.2	*BCL2L14*	Intron	T	0.50	0.86	0.81-0.91	1.8E-06
rs2106261	*ZFHX3*	rs12497228	3q29	*XXYLT1*	Intergenic	C	0.73	0.84	0.79-0.90	1.8E-06
rs2106261	*ZFHX3*	rs4677772	3q29	*XXYLT1*	Intergenic	A	0.74	0.84	0.78-0.90	1.9E-06
rs2106261	*ZFHX3*	rs2285117	22q13.31	*ARHGAP8*	Intron	T	0.25	0.83	0.77-0.90	2.0E-06

AF SNP		Interacting SNP						Interaction effects		
SNP	Closest gene	SNP	Locus	Closest gene	Location	Coding allele	CAF	OR[+]	95% CI[*]	*P* value
rs2106261	*ZFHX3*	rs2043264	3q29	*XXYLT1*	Intergenic	T	0.27	1.19	1.11-1.27	2.0E-06
rs2106261	*ZFHX3*	rs6874869	5q34	*TENM2*	Intergenic	T	0.90	0.78	0.70-0.86	2.2E-06
rs3807989	*CAV1*	rs3802477	9q22.33	*GABBR2*	Intron	T	0.95	0.74	0.66-0.83	3.6E-07
rs3807989	*CAV1*	rs2327995	6p22.3	*ATXN1*	Intron	T	0.73	0.87	0.82-0.92	4.3E-07
rs3807989	*CAV1*	rs17669102	6p22.3	*ATXN1*	Intron	T	0.26	1.15	1.09-1.21	7.0E-07
rs3807989	*CAV1*	rs17669356	6p22.3	*ATXN1*	Intron	T	0.79	0.87	0.82-0.92	2.2E-06
rs3807989	*CAV1*	rs3793100	6p22.3	*ATXN1*	Intron	A	0.21	1.15	1.09-1.22	3.8E-06
rs3807989	*CAV1*	rs4841108	8p23.1	*PPP1R3B*	Intergenic	A	0.33	1.15	1.08-1.23	5.9E-06
rs3807989	*CAV1*	rs447656	21q22.3	*ADARB1*	Intron	T	0.10	1.21	1.11-1.31	6.0E-06
rs3807989	*CAV1*	rs12916788	15q22.31	*MEGF11*	Intron	T	0.68	1.13	1.07-1.20	6.1E-06
rs3807989	*CAV1*	rs11631331	15q22.31	*MEGF11*	Intron	C	0.63	1.12	1.07-1.18	7.6E-06
rs3807989	*CAV1*	rs12903154	15q22.31	*MEGF11*	Intron	T	0.37	0.89	0.85-0.94	8.0E-06
rs3903239	*PRRX1*	rs288451	10q22.3	*LINC00595*	Intergenic	T	0.07	1.33	1.19-1.49	9.0E-07
rs3903239	*PRRX1*	rs7910940	10p14	*GATA3*	Intergenic	T	0.37	1.13	1.07-1.19	1.7E-06
rs3903239	*PRRX1*	rs7082115	10p14	*GATA3*	Intergenic	C	0.63	0.89	0.84-0.93	1.7E-06
rs3903239	*PRRX1*	rs10905431	10p14	*GATA3*	Intergenic	T	0.37	1.13	1.07-1.19	2.1E-06
rs3903239	*PRRX1*	rs4738374	8q21.11	*STAU2*	Intron	T	0.52	0.89	0.85-0.94	3.7E-06
rs3903239	*PRRX1*	rs4737384	8q21.11	*STAU2*	Intron	A	0.48	1.12	1.07-1.18	4.0E-06
rs3903239	*PRRX1*	rs13271172	8q21.11	*STAU2*	Intron	C	0.48	1.12	1.07-1.18	4.4E-06
rs3903239	*PRRX1*	rs844429	10q22.3	*LINC00595*	Intergenic	T	0.92	0.81	0.74-0.89	4.5E-06
rs3903239	*PRRX1*	rs149624	10q22.3	*LINC00595*	Intergenic	A	0.08	1.23	1.13-1.35	4.6E-06
rs3903239	*PRRX1*	rs6472783	8q21.11	*STAU2*	Intron	T	0.58	0.89	0.85-0.94	5.8E-06
rs4400058	*PITX2_3*	rs733804	17p13.1	*GAS7*	Intron	A	0.35	1.22	1.12-1.33	3.0E-06
rs4400058	*PITX2_3*	rs9380704	6p21.2	*ZFAND3*	Intergenic	T	0.27	1.22	1.12-1.33	5.7E-06
rs4400058	*PITX2_3*	rs4142102	6p21.2	*ZFAND3*	Intergenic	A	0.27	1.22	1.12-1.33	7.1E-06
rs4400058	*PITX2_3*	rs259693	6p21.2	*ZFAND3*	Intron	C	0.72	0.82	0.75-0.89	7.5E-06
rs4400058	*PITX2_3*	rs12523829	6p21.2	*ZFAND3*	Intergenic	A	0.26	1.22	1.12-1.33	8.3E-06
rs4400058	*PITX2_3*	rs9349051	6p21.2	*ZFAND3*	Intergenic	A	0.26	1.22	1.12-1.33	8.8E-06
rs4400058	*PITX2_3*	rs6697414	1p32.3	*SSBP3*	Intron	A	0.26	0.78	0.70-0.87	1.1E-05
rs4400058	*PITX2_3*	rs6484478	11p14.1	*ARL14EP*	Intergenic	A	0.30	1.22	1.12-1.33	1.2E-05
rs4400058	*PITX2_3*	rs9470723	6p21.2	*ZFAND3*	Intron	T	0.28	1.21	1.11-1.32	1.2E-05
rs4400058	*PITX2_3*	rs4711522	6p21.2	*ZFAND3*	Intron	A	0.73	0.83	0.76-0.90	1.2E-05
rs4642101	*CAND2*	rs10868854	9q21.11	*TRPM3*	Intron	A	0.54	0.88	0.84-0.93	3.7E-06
rs4642101	*CAND2*	rs10780951	9q21.11	*TRPM3*	Intron	T	0.46	1.13	1.07-1.19	4.6E-06
rs4642101	*CAND2*	rs2189325	7q31.1	*IMMP2L*	Intron	A	0.07	0.78	0.70-0.87	5.7E-06
rs4642101	*CAND2*	rs11657095	17q22	*MSI2*	Intron	A	0.15	1.18	1.10-1.27	8.8E-06
rs4642101	*CAND2*	rs17834063	17q22	*MSI2*	Intron	A	0.85	0.84	0.78-0.91	9.6E-06
rs4642101	*CAND2*	rs1374110	2q22.1	*THSD7B*	Intergenic	T	0.83	1.18	1.10-1.28	1.3E-05
rs4642101	*CAND2*	rs2079916	4q12	*LOC255130*	Promoter	A	0.35	1.13	1.07-1.19	1.4E-05
rs4642101	*CAND2*	rs2090174	7p21.3	*PER4*	Intergenic	A	0.26	0.86	0.80-0.92	1.5E-05

AF SNP		Interacting SNP						Interaction effects		
SNP	Closest gene	SNP	Locus	Closest gene	Location	Coding allele	CAF	OR[+]	95% CI[*]	*P* value
rs4642101	*CAND2*	rs6554438	4q12	*LOC255130*	Intergenic	A	0.35	1.13	1.07-1.19	1.6E-05
rs4642101	*CAND2*	rs913030	10q26.11	*NANOS1*	Intergenic	C	0.55	0.89	0.84-0.94	1.7E-05
rs6490029	*CUX2*	rs9874287	3p26.2	*LRRN1*	Intergenic	T	0.32	0.86	0.81-0.92	3.2E-06
rs6490029	*CUX2*	rs6011263	20q13.33	*LINC00659*	Promoter	T	0.86	0.82	0.76-0.89	4.0E-06
rs6490029	*CUX2*	rs2642099	14q21.3	*RPL10L*	Intergenic	T	0.09	1.26	1.14-1.39	4.7E-06
rs6490029	*CUX2*	rs6011258	20q13.33	*LINC00659*	Intergenic	A	0.86	0.82	0.76-0.89	4.8E-06
rs6490029	*CUX2*	rs4234126	2q37.3	*ANKMY1*	Intron	T	0.73	0.86	0.80-0.91	4.8E-06
rs6490029	*CUX2*	rs6011255	20q13.33	*LINC00659*	Intergenic	T	0.14	1.21	1.12-1.32	5.1E-06
rs6490029	*CUX2*	rs856945	20q13.33	*LINC00659*	3_UTR	A	0.86	0.83	0.76-0.90	5.2E-06
rs6490029	*CUX2*	rs856954	20q13.33	*LINC00659*	Intron	T	0.86	0.83	0.76-0.90	5.9E-06
rs6490029	*CUX2*	rs10198074	2q37.3	*ANKMY1*	Intron	T	0.73	0.86	0.80-0.92	6.2E-06
rs6490029	*CUX2*	rs13083844	3p26.2	*LRRN1*	Intergenic	T	0.68	1.16	1.09-1.24	7.0E-06
rs6666258	*KCNN3*	rs4895671	6q24.3	*EPM2A*	Intergenic	A	0.94	0.77	0.69-0.86	2.0E-06
rs6666258	*KCNN3*	rs1972364	6q24.3	*EPM2A*	Intergenic	A	0.06	1.30	1.17-1.45	2.0E-06
rs6666258	*KCNN3*	rs1972365	6q24.3	*EPM2A*	Intergenic	A	0.06	1.30	1.17-1.44	2.1E-06
rs6666258	*KCNN3*	rs4896791	6q24.3	*EPM2A*	Intergenic	T	0.94	0.77	0.69-0.86	2.5E-06
rs6666258	*KCNN3*	rs4895672	6q24.3	*EPM2A*	Intergenic	A	0.06	1.29	1.16-1.44	2.5E-06
rs6666258	*KCNN3*	rs1890200	6q24.3	*EPM2A*	Intergenic	A	0.06	1.30	1.16-1.44	2.6E-06
rs6666258	*KCNN3*	rs2048236	6q24.3	*EPM2A*	Intergenic	A	0.06	1.31	1.17-1.47	2.6E-06
rs6666258	*KCNN3*	rs4492209	6q24.3	*EPM2A*	Intergenic	T	0.06	1.29	1.16-1.44	2.6E-06
rs6666258	*KCNN3*	rs7751268	6q24.3	*EPM2A*	Intergenic	A	0.94	0.77	0.69-0.86	2.9E-06
rs6666258	*KCNN3*	rs7773601	6q24.3	*EPM2A*	Intergenic	A	0.06	1.31	1.17-1.47	3.1E-06
rs6817105	*PITX2*	rs4747672	10p11.23	*SVIL*	Intron	A	0.84	0.81	0.73-0.88	3.3E-06
rs6817105	*PITX2*	rs11597820	10p11.23	*SVIL*	Intron	A	0.84	0.81	0.74-0.89	4.5E-06
rs6817105	*PITX2*	rs10965244	9p21.3	*CDKN2B*	Intergenic	A	0.78	0.78	0.70-0.86	4.5E-06
rs6817105	*PITX2*	rs4747675	10p11.23	*SVIL*	Intron	C	0.79	0.82	0.75-0.89	5.3E-06
rs6817105	*PITX2*	rs7031458	9q21.32	*RASEF*	Intergenic	T	0.32	1.18	1.10-1.27	8.4E-06
rs6817105	*PITX2*	rs7676960	4p15.33	*LOC152742*	Intergenic	T	0.45	0.86	0.80-0.92	8.4E-06
rs6817105	*PITX2*	rs7694051	4p15.33	*LOC152742*	Intergenic	T	0.55	1.17	1.09-1.25	9.6E-06
rs6817105	*PITX2*	rs7901774	10p11.23	*SVIL*	Intron	T	0.17	1.22	1.12-1.34	9.9E-06
rs6817105	*PITX2*	rs2012160	2q37.3	*ANO7*	Intron	T	0.72	0.83	0.76-0.90	9.9E-06
rs6817105	*PITX2*	rs967559	2q37.3	*ANO7*	Intron	C	0.28	1.21	1.11-1.31	1.0E-05
rs6838973	*PITX2_4*	rs2634073	4q25	*PITX2*	Intergenic	T	0.19	0.86	0.81-0.92	3.0E-06
rs6838973	*PITX2_4*	rs2634071	4q25	*PITX2*	Intergenic	T	0.19	0.87	0.81-0.92	3.8E-06
rs6838973	*PITX2_4*	rs2231224	3q27.1	*AP2M1*	Synonymous	A	0.19	0.86	0.81-0.92	7.9E-06
rs6838973	*PITX2_4*	rs7614381	3q27.1	*ABCF3*	Intron	T	0.20	0.86	0.81-0.92	1.0E-05
rs6838973	*PITX2_4*	rs8478	3q27.1	*AP2M1*	Synonymous	T	0.19	0.87	0.81-0.92	1.0E-05
rs6838973	*PITX2_4*	rs136769	22q13.32	*C22orf34*	Intergenic	T	0.10	1.32	1.17-1.49	1.0E-05
rs6838973	*PITX2_4*	rs17741418	14q31.3	*GALC*	Intergenic	A	0.05	0.75	0.67-0.86	1.0E-05
rs6838973	*PITX2_4*	rs10867910	9q21.32	*RASEF*	Intergenic	T	0.33	0.88	0.83-0.93	1.1E-05

AF SNP		Interacting SNP						Interaction effects		
**SNP**	**Closest gene**	**SNP**	**Locus**	**Closest gene**	**Location**	**Coding allele**	**CAF**	**OR[+]**	**95% CI[*]**	***P* value**
rs6838973	*PITX2_4*	rs10867909	9q21.32	*RASEF*	Intergenic	A	0.67	1.13	1.07-1.20	1.1E-05
rs6838973	*PITX2_4*	rs2723300	4q25	*PITX2*	Intergenic	A	0.32	0.89	0.84-0.94	1.4E-05
rs7164883	*HCN4*	rs4980345	15q25.3	*SLC28A1*	Intron	T	0.06	1.44	1.27-1.65	4.3E-08
rs7164883	*HCN4*	rs12591784	15q25.3	*SLC28A1*	Intron	A	0.94	0.69	0.61-0.79	5.0E-08
rs7164883	*HCN4*	rs16974622	15q25.3	*SLC28A1*	Intron	A	0.94	0.70	0.61-0.80	1.0E-07
rs7164883	*HCN4*	rs12438877	15q25.3	*SLC28A1*	Intron	A	0.06	1.46	1.26-1.68	1.8E-07
rs7164883	*HCN4*	rs12440771	15q25.3	*SLC28A1*	Intron	T	0.94	0.69	0.60-0.79	1.9E-07
rs7164883	*HCN4*	rs11728305	4q12	*SRD5A3*	Intergenic	T	0.71	0.82	0.76-0.89	7.9E-07
rs7164883	*HCN4*	rs7341756	9q31.2	*KLF4*	Intergenic	A	0.09	1.36	1.20-1.56	3.9E-06
rs7164883	*HCN4*	rs12554275	9q31.2	*KLF4*	Intergenic	C	0.90	0.74	0.65-0.84	4.2E-06
rs7164883	*HCN4*	rs11887070	2q36.1	*KCNE4*	Intergenic	T	0.88	0.79	0.71-0.87	7.4E-06
rs7164883	*HCN4*	rs11914048	22q13.31	*PARVB*	Intron	T	0.77	0.82	0.74-0.89	1.0E-05

Acknowledgement

The study was supported by the German Federal Ministry of Education and Research (BMBF) in the context of the German National Genome Research Network (NGFN), the German National Competence network on atrial fibrillation (AFNET), the Leducq Foundation (07-CVD 03), the D.W. Reynolds Foundation Clinical Cardiovascular Research Center at Johns Hopkins University, and the Bioinformatics for the Functional Analysis of Mammalian Genomes program (BFAM) by grants to Stefan Kääb (01GS0499, 01GI0204, 01GS0838). The KORA study was initiated and financed by the Helmholtz Zentrum München – German Research Center for Environmental Health, which is funded by the German Federal Ministry of Education and Research (BMBF) and by the State of Bavaria. Furthermore, KORA research was supported within the Munich Center of Health Sciences (MC-Health), Ludwig-Maximilians-Universität, as part of LMUinnovativ. Dr. Sinner is supported by the German Heart Foundation.

The Atherosclerosis Risk in Communities Study is carried out as a collaborative study supported by National Heart, Lung, and Blood Institute contracts (HHSN268201100005C, HHSN268201100006C, HHSN268201100007C, HHSN268201100008C, HHSN268201100009C, HHSN268201100010C, HHSN268201100011C, and HHSN268201100012C,), R01HL087641, R01HL59367, R01HL086694, and R01HL111314; National Human Genome Research Institute contract U01HG004402; and National Institutes of Health contract HHSN268200625226C. The authors thank the staff and participants of the ARIC study for their important contributions. Infrastructure was partly supported by Grant Number UL1RR025005, a component of the National Institutes of Health and NIH Roadmap for Medical Research.

This CHS research was supported by NHLBI contracts N01-HC-85239, N01-HC-85079, N01-HC-85080, N01-HC-85081, N01-HC-85082, N01-HC-85083, N01-HC-85084, N01-HC-85085, N01-HC-85086; N01-HC-35129, N01 HC-15103, N01 HC-55222, N01-HC-75150, N01-HC-45133, HHSN268200800007C,

HHSN268201200036C and NHLBI grants HL080295, HL087652, HL103612, HL105756, HL120393 with additional contribution from NINDS. Additional support was provided through AG-023629, AG-15928, AG-20098, and AG-027058 from the NIA. See also http://www.chs-nhlbi.org/pi.htm.

The Cleveland Clinic AF study was supported by NIH grants to Drs. Chung, Barnard, Smith, and Van Wagoner (R01HL090620, R01HL111314), an NIH/NCRR CTSA grant (1UL-RR024989), Heart and Vascular Institute Philanthropic grant, Department of Cardiovascular Medicine, Cleveland Clinic (Chung), and a Leducq Fondation grant 07-CVD-03 (Van Wagoner).

The Framingham Heart Study is conducted and supported by the NHLBI in collaboration with Boston University (Contract No. N01-HC-25195; HHSN268201500001I). This project also was supported by NIH grants to Drs. Ellinor, Benjamin, and Lunetta (1RO1HL092577) and Benjamin and Ellinor (1R01HL128914).

LURIC was supported by the 7th Framework Program (AtheroRemo, grant agreement number 201668 and RiskyCAD, grant agreement number 305739) of the EU.

The PREVEND study is supported by the Dutch Kidney Foundation (grant E0.13), the National Institutes of Health (grant 2R01LM010098), The Netherlands organization for health research and development (NWO-Groot grant 175.010.2007.006, ZonMw grant 90.700.441), and the Dutch Inter University Cardiology Institute Netherlands (ICIN), and the Netherlands Heart Foundation (grant NHS2010B280). Dr. M. Rienstra is supported by a grant from the Netherlands Organization for Scientific Research (Veni grant 016.136.055). There are no relations with industry.

The PROSPER study was supported by an investigator initiated grant obtained from Bristol-Myers Squibb. Prof. Dr. J. W. Jukema is an Established Clinical Investigator of the Netherlands Heart Foundation (grant 2001 D 032). Support for genotyping was provided by the seventh framework program of the European commission (grant 223004) and by the Netherlands Genomics Initiative (Netherlands Consortium for Healthy Aging grant 050-060-810).

SHIP is part of the Community Medicine Research net of the University of Greifswald, Germany, which is funded by the Federal Ministry of Education and Research (grants no. 01ZZ9603, 01ZZ0103, and 01ZZ0403), the Ministry of Cultural Affairs as well as the Social Ministry of the Federal State of Mecklenburg-West Pomerania, and the network 'Greifswald Approach to Individualized Medicine (GANI_MED)' funded by the Federal Ministry of Education and Research (grant 03IS2061A). Genome-wide data have been supported by the Federal Ministry of Education and Research (grant no. 03ZIK012) and a joint grant from Siemens Healthcare, Erlangen, Germany and the Federal State of Mecklenburg-West Pomerania. The University of Greifswald is a member of the 'Center of Knowledge Interchange' program of the Siemens AG and the Caché Campus program of the InterSystems GmbH.

The generation and management of GWAS genotype data for the Rotterdam Study is supported by the Netherlands Organisation of Scientific Research NWO Investments (nr. 175.010.2005.011, 911-03-012). This study is funded by the Research Institute for Diseases in the Elderly (014-93-015; RIDE2), the Netherlands Genomics Initiative (NGI)/Netherlands Organisation for Scientific Research (NWO) project nr. 050-060-810. The Rotterdam Study is supported by the Erasmus MC and Erasmus University Rotterdam; the Netherlands Organisation for Scientific Research (NWO); the Netherlands Organisation for Health Research and Development (ZonMw); the Research Institute for Diseases in the Elderly (RIDE);

the Netherlands Genomics Initiative (NGI); the Ministry of Education, Culture and Science; the Ministry of Health Welfare and Sport; the European Commission (DG XII); and the Municipality of Rotterdam.

Dr. Ellinor is supported by grants from the National Institutes of Health (HL104156, HL105780, HL065962). Dr. Ellinor is also supported by an Established Investigator Award from the American Heart Association (13EIA14220013) and by support from the Fondation Leducq (14CVD01). Dr. Lubitz is supported by NIH grants K23HL114724, and a Doris Duke Charitable Foundation Clinical Scientist Development Award 2014105.
